# The potential of blue lupins as a protein source, in the diets of laying hens

**DOI:** 10.1016/j.vas.2016.11.004

**Published:** 2016-11-29

**Authors:** Michael R.F. Lee, Sarah Parkinson, Hannah R. Fleming, Vince J. Theobald, Dave K. Leemans, Tony Burgess

**Affiliations:** a Institute of Biological, Environmental and Rural Sciences, Gogerddan Campus, Aberystwyth University, Aberystwyth, Ceredigion SY23 3EB, UK; b Birchgrove Eggs, Trawscoed, Aberystwyth SY23 4AJ, UK

**Keywords:** Layer diet, Dietary protein, Blue lupin, Soya, *Lupinus angustifolius*

## Abstract

Layers diets typically contain 15–20% soya due to its high crude protein content (ca. 36%). Reliance on soya for protein can result in large increases in cost of feed due to the law of supply and demand as a global commodity. Lupin grains have high protein content (35–40%) but previous experience with white lupins has shown toxic effects in poultry due to high levels alkaloids and poor performance due to anti-nutritional Non-starch polysaccharides (NSP). Here blue lupins either processed or whole were trialled for their potential as a protein source. Point of lay chickens (64) at 16 weeks of age were weighed and allocated to 16 coops of four hens. Coops, as the experimental unit, were randomly allocated to four treatments: layers mash with soya (Control); or layers mash with 150 g of lupin/kg diet with the lupin either: whole (Whole); dehulled (Dehulled) or dehulled + a solid state fermentation enzyme extract (SSF; 150 g/tonne DM). All diets were ground and formulated to be balanced for energy, crude protein and essential amino acids using NIRS. No difference in growth rate, final hen weight, DM and water intake, eggs per day, mean egg weight, yellowness of yolk or chroma was found between treatments. There was a trend (P<0.1) for the SSF treatment to produce less heavy shells and a significant effect for the lupin treatments to have redder yolks (P<0.001). Fecal DM and bacterial counts were not different and there was no sign of enteritis or intestinal tissue hyperplasia from hen autopsies. Inclusion of blue lupins in the diet of laying hens at a rate of 150 g/kg DM resulted in no adverse effects in production or hen health and could be used as part of a balanced ration with inclusion of NSP degrading enzymes to reduce reliance on soya protein.

## 1 Introduction

The reliance of soya (*Glycine max*) as a protein source in the diets of monogastric (pigs and poultry) and ruminant livestock raises sustainability issues in terms of both the environment and economically. The main global producers of soya are the Americas (particularly USA, Argentina and Brazil). In the EU the transportation of soya over 5000 miles has serious environmental implications associated with Carbon emissions and life cycle analysis of the resultant animal product (Eisler et al., 2014). For example, the hectares abroad for feeding EU livestock can undermine food and land security in the exporting countries and off-balance natural nutrient cycles. In Brazil alone an area of 29.5 million hectares is devoted to growing soya (www.agrural.com.br). This crop has replaced thousands of square miles of small farms, whilst its highly mechanized cultivation has contributed to unemployment and rural migration. In addition, the impact of land use change and deforestation have further significant impacts on Carbon balance and biodiversity loss as the earth surpasses its safe operating boundaries (Rockström et al., 2009).

Economically the huge demand for soya makes the commodity fluctuate on the global stock markets, making the price of animal feed and ingredients a significant challenge for producers and feed processors driven by the laws of supply and demand, respectively. Furthermore, the demand for soya from other industries such as biofuel is set to increase as the EU has high biofuel consumption targets which will be partly met by importing soya oil from Argentina and Brazil (Knight, 2010). In addition to biofuel production, food production is also competing with the growing of biomass crops, which in turn has pushed global food prices such as soya even higher.

Lupin species (*Lupinus spp.*) have high crude protein content (comparable with soya) and may offer a solution for a more sustainable alternative to soya. Although, like most plant protein sources, methionine contents are low and supplementation of synthetic amino acids will be necessary to optimise poultry performance. Of the 200 + species of lupin, five species have been identified as suitable for cultivation as high-protein crops (*L. albus, L. angustifolius, L. luteus, L. mutabilis* and *L. polyphilu*; Gladstones, 1998). Early trials with white lupins (*L. albus*) in broilers were disappointing due to anti-nutritional factors (Oliver and Jonker, 1997, Olkowski et al., 2001, Wiryawan and Dingle, 1999). However, in a review by Van Barneveld (1999) it was concluded that high levels (300–400 g/kg DM) cold be included but levels are often restricted due to problems associated with excess moisture in the excreta. Lupins have unique carbohydrate properties characterised by negligible levels of starch, high levels of soluble and insoluble Non-starch polysaccharide (NSP), and high levels of raffinose oligosaccharides, all of which can affect the utilization of energy and the digestion of other nutrients in the diet. NSP; predominately polymeric galactan of D-galactose, L-arabinose, L-rhamnose and galacturonic acid; Van Barneveld (1999); Brillouet and Riochet, 1983; Knudsen, 2014) levels are twice as high as in other protein-rich legumes. As there are no endogenous NSP-degrading enzymes present in the avian digestive system high NSP has been shown to result in reduced feed intake, digestibility, increased water intake and sticky/wet feces which can increase the chances of bacterial load in the poultry house (Naveed et al., 1999). Lupins also contain low levels (0.003%) of bitter-tasting and potentially toxic alkaloids (Carey & Wink, 1993).

To over-come the anti-nutritional properties of lupins plant breeding programmes have selected cultivars with almost zero alkaloid content and current lupin cultivars are largely alkaloid free (Nalle et al., 2011). The removal of NSP through breeding has been more challenging, however the use of exogenous enzymes such as exo-galactanase incorporated into the feed has been shown to improve the gastro-intestinal digestibility of NSP and broiler performance (Steenfeldt et al., 2003). The removal of such constraints has seen an increase in the cultivation of lupins in parts of the world e.g. Australia and Southern Europe, whereas their use has been limited in Northern Europe due to difficult growing conditions and a short season. The development of blue (*L. angustifolius*) and yellow (*L. luteus*) lupin cold resistant varieties (Christiansen & Jornsgard, 2002) along with the development of agronomy for these species (Jones et al., 2005) has led to an opportunity to test the feasibility of ‘home-grown’ lupin to supply high quality protein into poultry rations.

Lupin meals have already been shown to be a useful ingredient in fish diets and are being used in commercial aquafeed in increasing quantities. The Australian blue lupin dominates world production driven by its use in aquaculture (Glencross & Hawkins, 2004). Typically, it is the dehulled kernel lupins that are used because of their greater nutritional value (Glencross et al., 2007). This study will investigate the potential of including blue lupin either whole or dehulled in addition to exogenous enzyme supplementation, to aid NSP degradation, versus a standard soya rich commercial layers ration as part of a nutrient balanced diet to laying hens.

## 2 Materials and methods

### 2.1 Animals, diets and experimental design

All procedures were carried out under the auspices of the Animal Scientific Procedures Act 1987 (ASPA) of Her Majesty's Britannic Government with the experiment approved by the Local Ethical Review Committee (LERC) of Aberystwyth University. All birds were closely monitored during the course of the experiment and a health check performed twice a day (09:00 and 17:00) with no occurrences of ill health recorded during the experiment. Severity limits were mild and not reached during the experiment.

Sixty-four point of lay (16 weeks of age) pullets (*Gallus gallus domesticus*; Bevan Brown) supplied by Joice and Hill Poultry Ltd (Eye, Peterborough, UK) with mean body weight of 1.4 ± 0.05 kg were allocated to 16 groups of four to give equal groups by weight. Each group was housed in a coop which acted as the experimental unit and in turn was randomly allocated to one of four treatments: Standard layer mash with soya (Control); Layers mash with 150 g/kg whole blue lupin (Whole); Layers mash with 150 g/kg dehulled blue lupin (Dehulled) or Layers mash with 150 g/kg dehulled blue lupin plus a solid state fermentation enzyme extract (150 g/tonne; Allzyme SSF) supplied by Alltech (Stamford, Lincolnshire, UK). SSF is a natural complex from Aspergillus Niger containing lactase and galactanase activity. The blue lupins were supplied by Soya UK (Southampton, Hampshire, UK) and dehulled by Alvan Blanch (Malmesbury, Wiltshire, UK). All diets were ground and formulated to be balanced for energy, crude protein, crude fiber, essential fats and amino acids as required for prelay and layers (Bovan Brown, Management Guide, Joice and Hill Poultry Ltd, Peterborough, UK) by Wynnstay Group (Llansaintffraid, Powys, UK; Table 1, Table 2) using a Unity Scientific Feed Analyzer (SpectraStar™ 2500) and a next generation high performance NIR analyzer for all feed ingredients (TRUE NIR™ spectrophotometer; Brookfield, CT, USA).

**Table 1:** Composition of the experimental diets (g/100 g DM).

	Control^a^	Whole^a^	Dehulled^a^	SSF^a^
Wheat (*Triticum aestivum*)	54.4	58.5	53.5	53.5
Wheatfeed	12.4	–	8.30	8.30
Corn (*Zea mays*)	5.85	5.00	5.00	5.00
Soya (*Glycine max*)	14.4	8.45	5.10	5.10
Whole Blue Lupin (*Lupinus angustifolius*)	–	15.0	–	–
Dehulled Blue Lupin	–	–	15.0	15.0
Salt (NaCl)	0.35	0.35	0.30	0.30
Limestone Granules	10.6	10.6	10.7	10.6
Sodium Bicarbonate	0.10	0.10	0.15	0.15
Di-calcium Phosphate	0.15	0.15	0.10	0.10
Rhodimet® AT88^b^	0.17	0.22	0.21	0.21
L-Lysine	0.08	0.14	0.11	0.11
L-Threonine	0.02	0.02	0.02	0.02
Layer Phytase and Xylanase Mix^c^	0.50	0.50	0.50	0.50
SSF Enzyme^d^	–	–	–	0.015
Vegetable oil	1.00	1.00	1.00	1.00

a Layers mash with soya (Control); layers mash with 150 g of lupin/kg diet with the lupin either: whole (Whole); dehulled (Dehulled) or dehulled + a solid state fermentation enzyme extract (SSF) (150 g/tonne DM).

b Adisseo, Antony, France.

c Trouw Nutrition, Ashbourne, Derbyshire, UK.

d Solid State Fermentation Enzyme Extract.

**Table 2:** Chemical composition of the formulated experimental diets (g/100 g).

	Control^a^	Whole^a^	Dehulled^a^	SSF^a^
Crude Protein	17.5	17.6	17.5	17.5
Metabolisable Energy (MJ/kg)	11.3	11.3	11.3	11.3
Oil	3.72	3.71	4.03	4.03
Crude Fibre	3.97	4.77	3.68	3.68
Organic Matter	86.1	86.4	86.3	86.3
Essential Fatty Acids	1.30	1.31	1.48	1.48
Lysine	0.78	0.76	0.76	0.76
Methionine	0.37	0.37	0.37	0.37
Threonine	0.57	0.55	0.55	0.55
Calcium	4.40	4.41	4.41	4.41
Available Phosphorus	0.30	0.30	0.30	0.30

a Layers mash with soya (Control); layers mash with 150 g of lupin/kg diet with the lupin either: whole (Whole); dehulled (Dehulled) or dehulled + a solid state fermentation enzyme extract (SSF) (150 g/tonne DM).

### 2.2 Animal housing and measurements

Coops allowed free access to a nesting area and perch with dropping board, water and feed troughs. The coops were floored with saw dust and sand. Hens were weighed individually at the start of the trial for allocation to groups and then again at the end of the growth phase at 22 weeks of age. During the laying phase (22 – 34 weeks of age) hens were weighed at the start of each week and averaged per coop. Eggs were collected from each coop daily. Conformation determined using egg charts supplied by AVLA egg inspectorate and weighed to determine total egg mass. On the first day of each week an egg selected at random was retained for further analysis from each coop. For the retained eggs, shell weight was determined by cracking the eggs – washing the shells, oven drying and weighing. The egg contents were used for yolk color analysis (as described below) and then frozen and freeze dried for total N determined by a micro-Kjeldahl technique using ‘Kjeltec’ equipment (Perstorp Analytical Ltd., Maidenhead, Berkshire, UK). Food DM and water intake were measured every day. A sample (200 g DM) of food was taken at the beginning of each week stored frozen and then freeze dried for total N analysis as above. Fecal collection (excreta) occurred at the start of each week by removing the excreta from the dropping board under the perches weighing and storing at −20 °C prior to analysis (Freeze dry matter and total N).

### 2.3 Egg yolk color analysis

Egg yolk color was investigated using a Minolta CR-200 Chroma meter (Konica-Minolta, Warrington, UK), calibrated to a standard white plate (Calibration Plate CR A43) to gain precise chromaticity. An 8 mm diameter measuring area, diffuse illumination, from a xenon (PXA) arc lamp, and 0° viewing angle gave precise measurements across the whole sample. The tip of the Chroma meter measuring head was placed flat against the surface of a Petri dish and yolk reflective color was determined from the average of three consecutive pulses from the optical chamber of the Chroma meter. Absoluteness of color was measured using L*a*b* co-ordinates (Dvorak et al., 2009).

### 2.4 Bacterial enumeration and autopsies

On the final week of the egg laying phase a sub-sample of feces from each coop was collected fresh and 10 g weighed into a test tube containing 90 mL maximum recovery diluent and serially diluted logarithmically four times. From each of these five serial dilutions, 100 µL was spread in triplicate onto plates containing Xylose lysine deoxycholate (XLD) agar (Sigma-Aldrich, Gillingham, Dorset, UK). Plates were incubated at 37 °C for 46 h and colonies counted for the identification of red colonies (*Salmonella spp., Shigella spp., Providencia spp.*) and yellow colonies (*Enterobacteriaceae*).

At the end of the 12 week egg laying phase a hen was selected at random from each coop, euthanized using a schedule 1 procedure as required under ASPA (neck dislocation) and a gut autopsy performed to determine gut health and any sign of disease such as gut enteritis. Photos were taken of the removed tracts and assessed on a 5 point scale for signs of disease with 1 healthy and 5 chronic disease. Measurements were also taken on gut size (length – duodenum to ceca).

### 2.5 Statistical analysis

The experimental unit was the coop and hen weight and analysed using a general analysis of variance (ANOVA). Bacterial counts were analysed using ANOVA of log_10_ transformed data, whereas hen performance and egg quality during the laying period were analysed using repeated measures ANOVA, with week as the time variant, diet as the treatment and blocked by coop number (Genstat 16.1; Lawes Agricultural Trust, Harpenden, Hertfordshire, UK).

## 3 Results

### 3.1 Hen performance

**Fig. 1:**
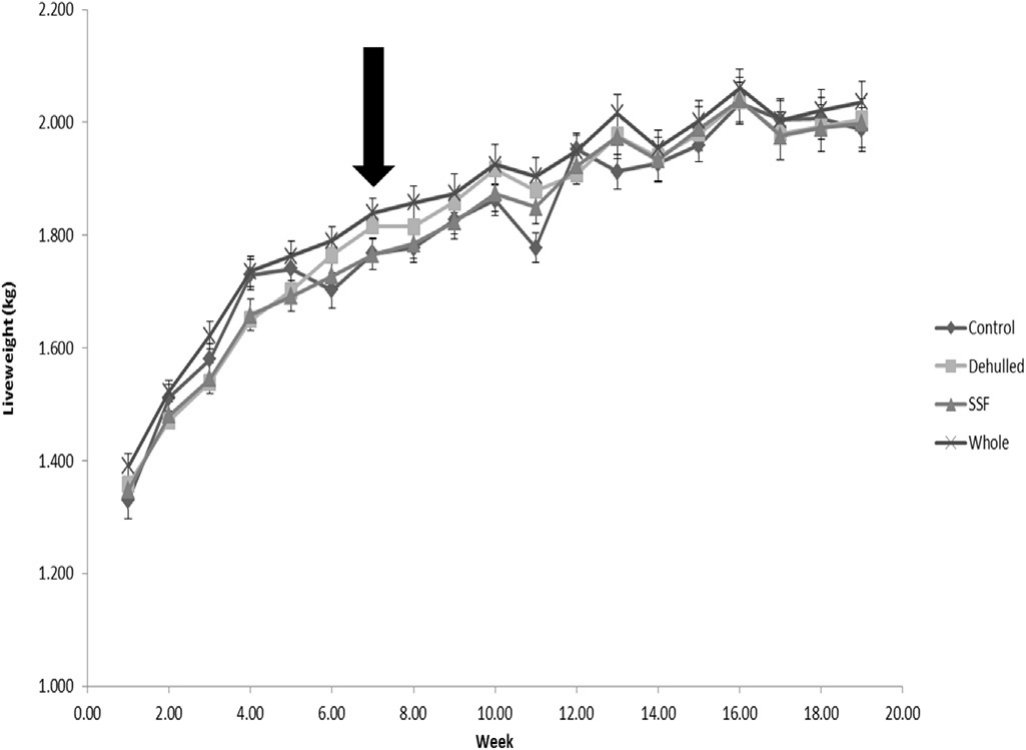
Live weight gain during the growth and egg laying phase of hens offered the four experimental diets. (Layers mash with soya (Control); layers mash with 150 g of lupin/kg diet with the lupin either: whole (Whole); dehulled (Dehulled) or dehulled + a solid state fermentation enzyme extract (SSF) (150 g/tonne DM); Arrow indicates the end of the growth phase).

Diets were formulated to be iso-nitrogenous and iso-energetic, and to provide the recommended level of essential amino acids and fatty acids (Table 1, Table 2). Live weight gain of the hens throughout the 18 week trial is reported in Fig. 1. No difference was observed as a mean across the growth and egg laying phase with mean weight at 34 weeks of age 2.0 ± 0.04 kg (Table 3). A slight decrease in live weight was observed for the control group at week 11 (P<0.05), but the following week this difference was lost with no subsequent difference throughout the experiment.

**Table 3:** Layers performance and egg quality during the 12 week egg laying phase.

	Control^a^	Whole^a^	Dehulled^a^	SSF^a^	s.e.d.	Significance
Bird weight at 34wks (kg)	1.98	2.04	2.01	2.00	0.040	NS
DM Intake (g/d)	142	132	133	133	5.23	NS
Water Intake (mL/d)	240	239	262	245	23.5	NS
Eggs per day	0.98	0.97	0.96	0.96	0.013	NS
Egg weight (g)	58.9	60.6	59.3	58.8	1.03	NS
Shell weight (g)	5.74^a^	5.97^a^	5.94^a^	5.64^b^	0.127	0.065
Yolk lightness (L)	60.7^b^	59.8^a^	59.6^a^	59.6^a^	0.18	0.001
Yolk yellowness (b*)	32.4	32.9	32.1	32.6	0.66	NS
Yolk redness (a*)	3.89^a^	5.74^b^	6.23^b^	6.29^b^	0.482	0.001
Yolk Chroma	32.7	33.5	32.7	33.2	0.66	NS
Fecal DM (%)	26.8	27.0	25.4	26.7	1.14	NS

a Layers mash with soya (Control); layers mash with 150 g of lupin/kg diet with the lupin either: whole (Whole); dehulled (Dehulled) or dehulled + a solid state fermentation enzyme extract (SSF) (150 g/tonne DM).

a Values within a row with different superscript differ significantly *P*<0.05.

b Values within a row with different superscript differ significantly *P*<0.05.

**Fig. 2:**
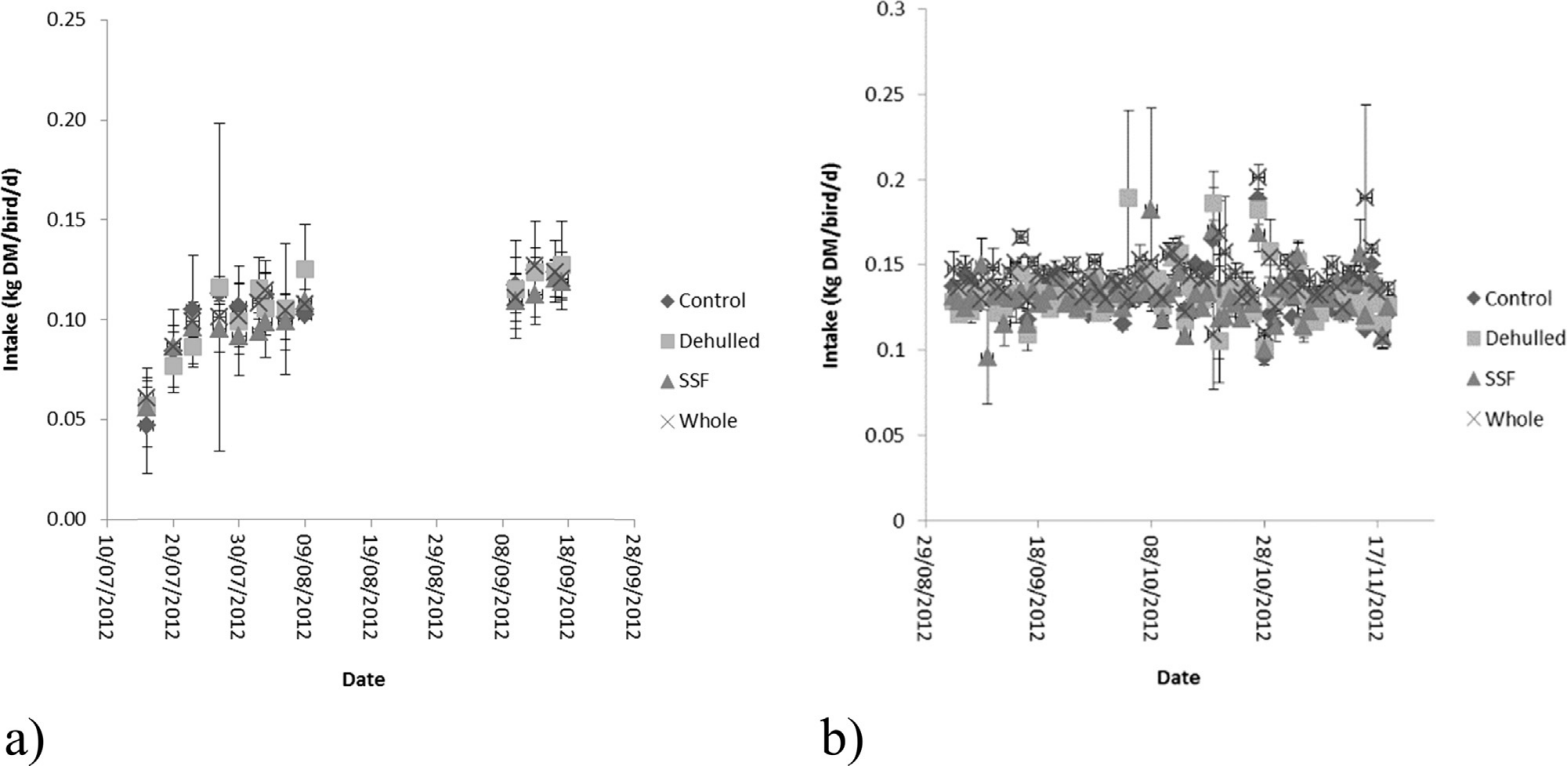
Daily dry matter intake of hens offered the four experimental diets. (a) Growth phase, (b) Laying phase (Layers mash with soya (Control); layers mash with 150 g of lupin/kg diet with the lupin either: whole (Whole); dehulled (Dehulled) or dehulled + a solid state fermentation enzyme extract (SSF) (150 g/tonne DM).

**Fig. 3:**
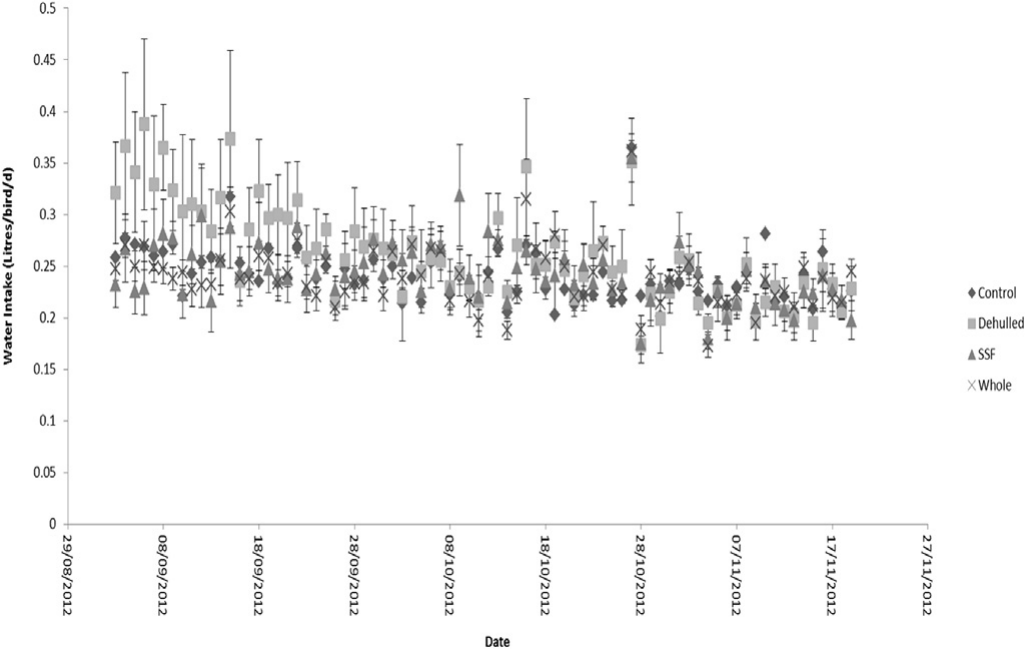
Daily water intake of the hens offered the four experimental diets during the egg laying phase (Layers mash with soya (Control); layers mash with 150 g of lupin/kg diet with the lupin either: whole (Whole); dehulled (Dehulled) or dehulled + a solid state fermentation enzyme extract (SSF) (150 g/tonne DM).

Daily dry matter intake during the growth and egg laying phases are reported in Fig. 2. Dry matter intake at the start of the experiment (age 16 weeks) was 60 ± 30.0 g/hen/d in the first week rising to 135 ± 5.2 g/hen/d as the hens entered the egg laying phase (age 22 weeks) and then remained relatively constant. Outliers were observed during the egg laying phase for Dehulled, SSF and Whole treatments, but were associated with large error values and were not indicative of any pattern for difference in dry matter intake during the experiment. Water intake for the egg laying phase is reported in Fig. 3. There was a trend (P<0.1) within the first week for higher intake on the Dehulled treatment, however the variance associated with these means was large and by the second week no difference was observed for any of the treatments with mean water intake 247 ± 23.5 mL/hen/d (Table 3).

**Fig. 4:**
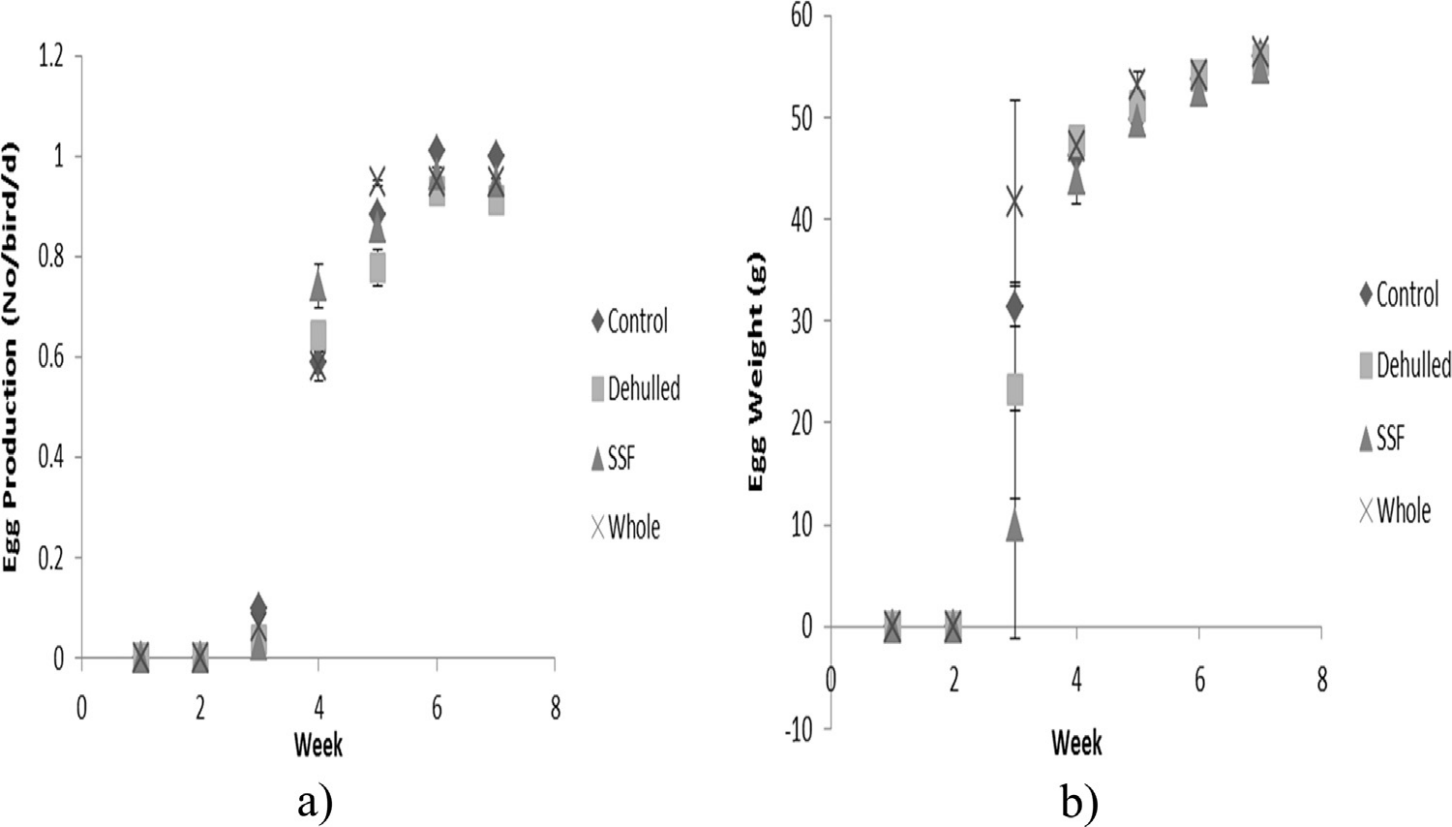
Daily egg production and weight of the hens offered the four experimental diets during the growth phase. (a) Production, (b) Weight (Layers mash with soya (Control); layers mash with 150 g of lupin/kg diet with the lupin either: whole (Whole); dehulled (Dehulled) or dehulled + a solid state fermentation enzyme extract (SSF) (150 g/tonne DM).

**Fig. 5:**
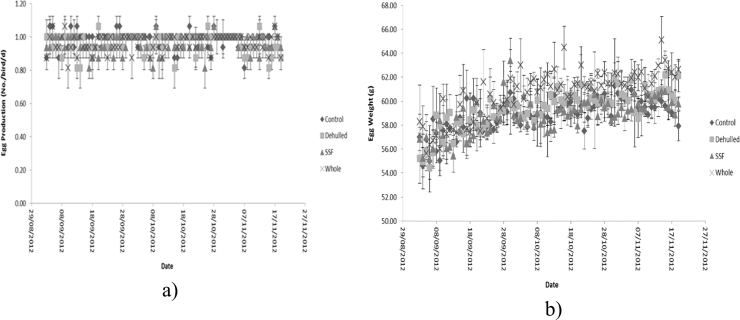
Daily egg production and weight of the hens offered the four experimental diets during the egg laying phase. (a) Production, (b) Weight (Layers mash with soya (Control); layers mash with 150 g of lupin/kg diet with the lupin either: whole (Whole); dehulled (Dehulled) or dehulled + a solid state fermentation enzyme extract (SSF) (150 g/tonne DM).

Daily egg production and respective egg weights during the growth phase are reported in Fig. 4. Treatment groups produced their first pullet eggs by week 3 (19 weeks of age) with large variations in egg weight with Whole ≥ Control ≥ Dehulled ≥ SSF. The following week the SSF treatment produced more eggs than the other treatments (P<0.05), of similar weights, whereas in the fifth week the Whole treatment produced the most eggs with the Dehulled treatment the least (P<0.05). At the end of the growth phase and during the egg laying phase mean egg production and egg weight across the four dietary treatments was not different and averaged 0.97 ± 0.01 eggs/hen/d and 59.4 ± 1.03 g, respectively (Table 3 and Fig. 5). Mean egg weight during the 12 week egg laying phase increased linearly (P<0.05) from 56.9 ± 4.01 g to 60.6 ± 1.91 g, (Fig. 5b) with no difference between treatments.

### 3.2 Egg quality

There was a trend (P=0.065) for the SSF treatment egg shells to be lighter than the other treatments 5.64 (SSF) vs. 5.89 (average of Control, Whole and Dehulled) g (Table 3). Yolk yellowness (b*) and chroma were not different between dietary treatments averaging 32.5 ± 0.66 and 33.0 ± 0.66, respectively. Lightness of the yolk was significantly higher on the Control diet versus all diets containing lupins 60.7 vs. 59.7 and likewise redness (a*) was significantly lower on the Control than the lupin containing diets 3.89 vs 6.09 (Table 3).

### 3.3 Nitrogen concentrations

Dietary N content of the Control diet was higher than the three lupin containing feeds (31.5 vs 29.2 g/kg DM). Fecal and Egg N were not different across the four dietary treatments averaging 52.2 ± 2.81 and 86.4 ± 0.12 g/kg DM, respectively (Table 4).

**Table 4:** Concentration of nitrogen: dietary, egg and fecal (g/kg DM).

	Control^a^	Whole^a^	Dehulled^a^	SSF^a^	s.e.d.	Significance
Dietary N	31.5^b^	29.3^a^	28.7^a^	29.5^a^	0.51	0.002
Fecal N	50.4	50.8	54.5	53.3	2.81	NS
Egg N	86.5	86.1	87.1	85.7	0.12	NS

a Layers mash with soya (Control); layers mash with 150 g of lupin/kg diet with the lupin either: whole (Whole); dehulled (Dehulled) or dehulled + a solid state fermentation enzyme extract (SSF) (150 g/tonne DM).

a Values within a row with different superscript differ significantly *P*<0.05.

b Values within a row with different superscript differ significantly *P*<0.05.

### 3.4 Hen health

**Fig. 6:**
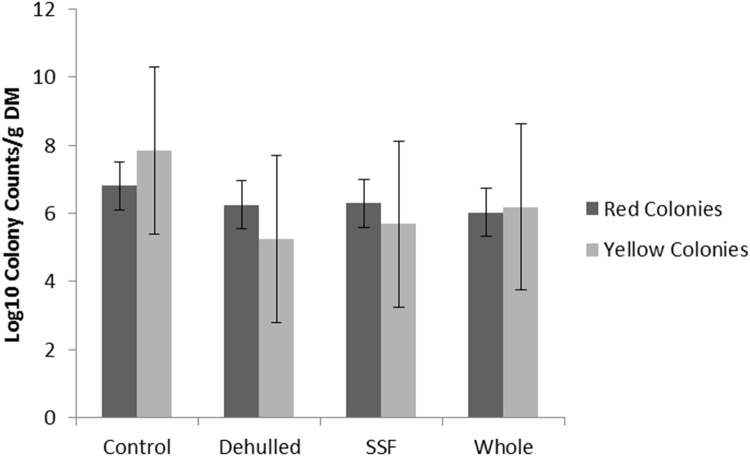
Bacterial colony counts from the feces of hens offered the four experimental diets for 20 weeks (Layers mash with soya (Control); layers mash with 150 g of lupin/kg diet with the lupin either: whole (Whole); dehulled (Dehulled) or dehulled + a solid state fermentation enzyme extract (SSF) (150 g/tonne DM).

Fecal dry matter was not different across experimental dietary treatments (mean 26.5 ± 1.14%; Table 3) with no difference in bacterial enumeration (Fig. 6). Gut autopsies performed on all hens scored 1 on the 5 point scale with no sign of enteritis or gut disease. Average length of the gastro-intestinal tract from duodenum to ceca was 93 ± 10.5 cm and was not different between treatments.

## 4 Discussion

### 4.1 Lupins and hen performance

Diets were formulated to be iso-nitrogenous, iso-energetic and provide all the required essential amino acids, and fatty acids as well as vitamins and minerals. This allowed the potential of blue lupin incorporation into the diet to be determined when balanced for nutrient provision. Previous researchers have highlighted the importance of balancing for methionine, lysine and threonine in diets containing lupin and special consideration was therefore given to balancing for these essential amino acids (Hammershoj & Steenfeldt, 2005).

Steenfeldt et al. (2003) reported that incorporation of blue lupin at 200 g/kg in broiler diets did not reduce feed intake compared to a control however incorporation significantly depressed weight gain and feed conversion efficiency. They also reported that the use of an exogenous enzyme extract, similar to SSF used in the current study (www.alltech.com/product/allzyme-ssf), containing lactase and galactanase activity significantly improved digestibility and weight gain. In the present study similar results in terms of feed intake were observed between the lupin and non-lupin containing diets but in contrast all diets produced similar growth rates, this may be related to the lower level of inclusion of blue lupin into the diet in the current study (150 g/kg) or a difference between broilers and layers to digest the lupin protein. Although Nalle et al. (2011) also reported positive results in terms of weight gain and feed intake with broilers at an inclusion rate of 200 g/kg of white lupin. Previous research when feeding blue lupins to layers has shown that at levels of lupin inclusion similar to the present study (147.8 g/kg) DM intake was not affected, although at higher levels (250 g/kg) intake was significantly reduced [18]. However, both levels of lupin inclusion in the study of Hammershoj and Steenfeldt, (2005) reduced live weight gain in agreement with the broiler study of Steenfeldt et al. (2003) and in contrast to the present study and Nalle et al. (2011). The level of NSP reported in the study of Steenfeldt et al. (2003) was higher than the average value determined by a global review of grain quality reported by Petterson (2004) (450 vs. 284 g/kg DM, respectively) and so indicates a wide variation in NSP content which will influence digestibility. The high NSP level reported by Steenfeldt et al. (2003) explains the positive impact of enzyme addition on digestibility and weight gain. SSF, used in the current study, is a solid state fermentation product of *Aspergillus niger* designed to aid with the degradation of NSP containing seven enzyme activities: α-amylase, β-glucanase, cellulose, pectinase, phytase, protease and xylanse (Cheng et al., 2005). However, in the current study all diets were also formulated with a Layer Phytase and Xylanase Mix (Table 1), as current commercial practice, which may have been sufficient to aid digestion of the NSP and also explain the lack of any further response through the addition of SSF. Similar results were found with the inclusion of yellow lupin (*Lupinus luteus*) into layers diets which also contained an exogenous enzyme extract and produced no difference in hen performance when matched for energy, protein and essential nutrient provision (Mackintosh et al., 2014). Hammershoj and Steenfeldt, (2005) did not use synthetic amino acids in their ration, as it was based on organic poultry, subsequently the higher level of lupin resulted in a lower intake of methionine, which may have resulted in reduced growth rates (Pourreza & Smith, 1988). This highlights an essential role of formulation to tackle NSP and amino acid balance in order to facilitate inclusion of more sustainable sources of protein such as lupin into the diets of hens.

Egg production and performance patterns were similar to those previously reported for laying hens at similar stages of lay (Hammershoj and Steenfeldt, 2005, Mackintosh et al., 2014, Prinsloo et al., 1992). In the present study there was no difference between Control, Dehulled, Whole or SSF as also reported by MacKintosh *et al.* (2014) when offering yellow lupins at the same inclusion rate (150 g/kg). Prinsloo et al. (1992) reported similar responses in egg production with white lupins, whereas other studies have shown a significant reduction in egg mass with lupin inclusion for reasons relating to NSP and amino acid bioavailability as discussed above (Mackintosh et al., 2014, Watkins and Mirosh, 1987, Perez-Maldonado et al., 1999).

A detailed analysis of the structural and non-structural carbohydrate composition of the whole, cotyledon and hull is given by Van Barneveld (1999) and Knudsen (2014) with total dietary fibre averaging 416 g/kg DM, predominately found in the hull (83% DM). The fibrous hull of the whole lupin has been reported to contribute to growth depression due to lower digestibility in poultry (Brenes et al., 1993; 2002) and aquaculture (Glencross et al., 2007), however in the current study Whole lupins performed as well as Dehulled lupins in terms of hen performance (live weight gain and egg production). The higher level of fibre in the whole lupins (Table 2) had no effect on dry matter intake or animal performance. Brenes et al. (1993) noted that the hull of lupins contains a high concentration of ADF (cellulose and lignin - 65%) which would be less digestible / indigestible to poultry, and in their study reduced performance over dehulled lupins. However, inclusion rates were significantly higher (50%) than in the current study and in that of MacKintosh *et al.* (2014) who also found no response for dehulled lupins as opposed to whole lupins. Mateos et al. (2011) reported that fibre within the diet of poultry at a low level (2–3%) can improve dietary performance which is comparable to the fibre level achieved in the current study (3.7–4.8%) and may explain the lack of response for the Dehulled vs. Whole comparison.

Although not significantly different over the course of the experiment, water intake tended to be higher for Dehulled as opposed to Whole or SSF for the first week of the laying phase. This may be attributable to the great bioavailability of the NSP to micro-organisms in the gastro-intestinal tract due to the removal of the ‘protective hull’ and lower level of degrading enzymes. Greater levels of available raffinose-oligosaccharides have been shown to promote an osmotic effect in the gastro-intestinal tract supporting the growth of bacterial populations and may reflect a greater thirst in the hens (Brenes et al., 1989). However, difference in water intake response was later lost in the second week and highly variable, which may reflect acclimatisation at different rates across hens. Unfortunately, water intakes were not taken during the growth phase and so cannot confirm whether this was a true response of hens acclimatising to the Dehulled lupin.

Although the diets were rationed to be iso-nitrogenous using NIRS the analysis of the feed showed a higher N content in the control diet than the lupin containing diets. The difference was numerically small, but was significant further highlighting the potential of lupin to be incorporated into the diet as no difference in performance was observed despite the lower N intake. Although a full N balance study was not performed here to determine N and OM digestibility, fecal N was not different between treatments.

### 4.2 Lupins and egg quality

Egg protein content (Egg N) was not effected by either of the treatments as it has been shown that the protein content of eggs is highly regulated at ca. 50% DM (Bologa et al., 2013). There was a trend for shell weight to be lower with the SSF treatment than the other treatments. SSF supplementation has been shown to improve P utilisation and in low P diets has improved hen performance (Cheng et al., 2005). Phosphate ions have an inhibitory effect on CaCO_3_ and bring shell formation to an end. Therefore, high levels of P in the blood will inhibit the mobilization of calcium from bone and could reduce Ca deposition for shell formation, although further research will be needed to determine whether SSF could improve P availability on a nutrient balanced diet to such an extent to influence Ca deposition into egg shell.

Yolk color is an important aspect of consumer acceptance with most of the consumers questioned in a European survey expressing a preference for darker redder yolks (Beardsworth & Hernandez, 2004). Egg yolk color is a balance between yellow and red, both of which are influenced by the carotenoids from the diet: lutein, zeaxanthin and apo-ester are associated with yellow and canthaxanthin, citraxanthin and astaxanthin are associated with red. Lupins in laying poultry diets previously have given contrasting results from: no effect (Quarantelli et al., 1993), decreased color (Vogt et al., 1987), increased yellowness (Hammershoj & Steenfeldt, 2005) to increased redness (Mackintosh et al., 2014). The current study agrees with the latter results of MacKintosh *et al.* (2014) where lupin inclusion significantly increased redness and subsequently decreased lightness. Mohamed and Rayas-Duarte (1995) reported a mixture of both red and yellow carotenoids associated with lupin kernels, which varied across species and cultivars, which helps explain the contrasting results reported on the effect of lupin on yolk color in the literature.

### 4.3 Lupins and hen health

Lupins have been reported to have significant effects on hen health including chronic signs of toxicity (Olkowski et al., 2005). Health issues with current low alkaloid lupin varieties appear to be associated with NSP content which is associated with decreased use of nutrients, increased digesta viscosity and enlargement of the intestines (Olkowski et al., 2005). Further health issues then arise as the viscosity of the digesta results in sticky faeces which can increase the chances of bacterial load in the poultry house (Naveed et al., 1999). In the current study no difference was found in fecal DM or bacterial load of the feces in the coops. Autopsy of the gastro-intestinal tract also revealed no difference in size or health with no sign of inflammation or intestinal tissue hyperplasia as previously reported in poultry offered lupin containing diets. These results mirror the animal performance result within the current study and indicate that lupins are suitable for inclusion into poultry rations when the diets are well balanced (energy, protein, essential amino acids, fatty acids, minerals and vitamins) and include sufficient exogenous enzymes to aid degradation of NSP.
